# Supplementary material for: Systematic Review of Actinomycetes in the Baijiu Fermentation Microbiome
Source: Foods. 2022 Nov 8;11(22):3551. doi: 10.3390/foods11223551 (PMC9689711; doi:10.3390/foods11223551)
Supplement: Supplementary file 1 [file foods-11-03551-s001.zip › foods-1987434-supplementary.pdf]

Table S1. Culture media used for isolation of actinomycetes

Medium	Formulation
Gause No. 1 medium (g/L)	Soluble starch 20 g, KNO_3 1 g, K_2HPO_4 0.5 g, $MgSO_4 \cdot 7H_2O$ 0.5 g, NaCl 0.5 g, $FeSO_4 \cdot 7H_2O$ 0.01 g, Agar 20 g, pH 7.4~7.6
Modified Gause No. 2 Medium (g/L)	Glucose 1 g, Peptone 0.5 g, Tryptone 0.3 g, Sodium chloride 0.5 g, Multivitamin 2.75 mg (Including 0.5 mg each for VB_1[1],VB_2, VB_3, VB_5, VB_6, 0 .25 mg for VB_7), Agar 20.0 g, pH 7.2-7.4
Beef extract peptone medium (g/L)	Peptone 10.0 g, Beef extract 3.0 g, NaCl 5.0, Agar 20.0 g, pH 7.4~7.6
GTY medium (g/L)	Glucose 1.0 g, tryptone 0.5 g, Yeast powder 2.0 g, $CaCO_3$ 1.0 g, agar 20.0 g, pH nature
ISP2 medium (g/L)	Yeast extract 4.0 g, Malt extract 10.0 g, Glucose 4.0 g, Agar 15.0 g, pH 7.3
R2A medium (g/L)	Yeast extract 0.5 g, Peptone 0.5 g, Acid hydrolysate of casein 0.5 g, Glucose 0.5 g, Soluble starch 0.5 g, Dipotassium phosphate 0.3 g, Anhydrous magnesium sulfate 0.024 g, Sodium pyruvate 0.3 g, Agar 15.0 g, pH 7.2
Situ-medium	Huangshui fluid 1 L, Agar powder 1.5% (mass fraction), Gellan gum 0.5% (mass fraction), pH nature
Solid state fermentation medium	Oat flour 50 g, Trace salt solution 1 mL (KNO_3 1%, KH_2PO_4 0.5%, $MgSO_4$ 0.5%, $FeSO_4$ 0.1%, NaOH 0.8%) (mass fraction), pH nature
Complete Inorganic Basal Medium	K_2HPO_4 500m g, $MgSO_4 \cdot H_2O$ 250m g, Na_2CO_3 100m g, FeEDTA 5m g, $(NH_4)_2CO_3$ 500m g, $CaCl_2$ 100m g, $Na_2SiO_3 \cdot 9H_2O$ 100m g, Trace salt solution 1mL, Agar 1.5g
YEME medium (g/L)	Glucose 10 g, Sucrose 100 g, Peptone 5 g, Malt extract 3 g, Yeast extract 3 g
Oat juice medium	Raw oat flakes 20 g, Soluble starch 10 g, Trace salt solution 1 mL ($FeSO_4$ 0.1%, $MgCl_2$ 0.1%, $ZnSO_4$ 0.1%) (mass fraction), pH nature
Modified Gause No.1 Medium	Soluble starch 20 g, NaCl 0.5 g, $K_2HPO_4 \cdot 3H_2O$ 0.5 g, $MgSO_4 \cdot 7H_2O$ 0.5 g, $FeSO_4 \cdot 7H_2O$ 0.01 g, Agar 20g (including 100mL sterile water extract of fermented grains, pit mud and DQ and 50000U nystatin) pH nature
Modified Gause No. 2 Medium	Glucose 10 g, Peptone 5 g, Tryptone 3 g, NaCl 5 g, Agar 20 g, Water 1 L, pH 7.2, Nutrient solution 10 mL, 1 mL (50 mg/mL) Nalidixic acid, 0.4 mL (20 g mg/mL) Amphotericin
GYM medium (g/L)	Glucose 4.0 g, Yeast extract 4.0 g, Malt extract 10.0 g, Calcium carbonate 2.0 g, Agar 12.0 g, Distilled water 1000.0 mL, pH 7.0-7.2
ISP3 medium	Oat flour 20.0 g, Trace element solution 1 mL (trace element solution: $FeSO_4 \cdot 7H_2O$ 0.01 g,

(g/L)	$MnCl_2·4H_2O$ 0.1 g, $ZnSO_4·7H_2O$ 0.1 g, distilled water 100 mL), Agar 15.0 g, pH 7.2
ISP4 medium (g/L)	Soluble starch 10.0 g, K_2HPO_4 1.0 g, $MgSO_4·7H_2O$ 1.0 g, NaCl 1.0 g, $(NH_4)_2SO_4$ 2.0 g, $CaCO_3$ 2.0 g, $FeSO_4·7H_2O$ 0.001 g, $MnCl_2·4H_2O$ 0.001 g, Agar 20.0 g, Distilled water 1000 mL, pH 7.0-7.2
ISP5 medium (g/L)	L-aspartic acid 1.0 g, Glycerol 10.0 g, K_2HPO_4 1.0 g, Trace element solution 1 mL (same as ISP3 medium), Agar 20.0 g, Distilled water 1000 mL, pH 7.0-7.2
Humic acid medium (g/L)	Humic acid 1.0 g, Na_2HPO_4 0.5 g, KCl 1.7 g, $MgSO_4·7H_2O$ 0.05 g, $FeSO_4·7H_2O$ 0.01 g, CaCl2 1.0 g, Vitamin mixture 3.75 mg (including VB1, VB2, VB3, VB5, VB6, inositol, 0.5 mg of p-aminobenzoic acid and 0.25 mg of vitamin H)
Starch-glycerol medium (g/L)	Starch 10 g, Glycerol 10 g, $(NH_4)_2SO_4$ 2 g, $CaCO_3$ 1 g, $MgSO_4·7H_2O$ 1.0 g, NaCl 1.0 g, Proline 10 g, Agar 12 g, pH 7
Casein medium (g/L)	$Na_2HPO_4·7H_2O$ 1.07 g, KH_2PO_4 0.36 g, Casein 4 g, $ZnCl_2$ 0.014 g, NaCl 1.2 g, $CaCl_2$ 0.002g，$MgSO_4·7H_2O$ 0.5 g, $FeSO_4$ 0.02 g, Agar 20 g pH nature
enriched medium	NaCl 1.0 g, K_2HPO_4 1.0 g, NH_4Cl 1.0 g, $MgCl_2$ 0.5 g, $NaNO_3$ 1.0 g, Glucose 2 g, (Dissolved in 500 mL distilled water, 20 g distiller's grains, 10 mL huangshui fluid, 6 g Daqu powder, 20 mL of last distillate), pH 6.5～7.0
Inorganic salt medium	$MgSO_4$ 0.5 g、KH_2PO_4 1.0 g、NH_4Cl 1.0 g、6.67 g/L $CaCl_2$ 3.0 mL、17 g/L $FeCl_3$ 3.0 mL、$FeSO_4·7H_2O$ 0.05 g、$NaNO_3$ 1.0 g、n-Propanol (sole carbon source)
Modified Gause No. 2 Medium	Glucose 1 g, Peptone 0.5 g, Tryptone 0.3 g, Sodium chloride 0.5 g, Multivitamin 2.75 m g, Agar 20.0 g, Distilled water 1 000 mL, pH 7.2~7.4
PDA medium (Percentage)	Potato dip powder 0.6%; Glucose 2%; Agar 2%, pH 6.0
broth medium (Percentage)	Beef extract 0.5%, Peptone 1%, Sodium chloride 1%, pH 7.2~7.5
GW1 medium (g/L)	Caseins 5 g, Mannitol1 g, $NaHCO_3$ 2 g, $CaCO_3$ 0.2 g, $(NH_4)_2SO_4$ 2 g, KNO_3 2 g, K_2HPO_4 1 g, $MgSO_4·7H_2O$ 2 g, $FeSO_4·7H_2O$ 0.02 g, KCl 0.01 g, ddH_2O 1 L；PH7.2-7.4
GMKA medium (g/L)	Caseins 0.5 g, Mannitol 1.5 g, KNO3 1.0 g, K_2HPO_4 0.5 g, $(NH_4)_2SO_4$ 2 g, $CaCO_3$ 0.5 g, Agar 15 g, ddH_2O 1 L
HV medium (g/L)	Humic acid 5 g, $CaCO_3$ 0.02 g, Na_2HPO_4 0.5 g, KCl 1.7 g, $FeSO_4·7H_2O$ 0.01 g, $MgSO_4·7H_2O$ 0.5 g, Agar 5 g, ddH_2O 1 L, pH 7.2-7.4
LSA medium (g/L)	Pancreatic digest of casein 10.0g，Yeast extract 5.0 g, Glucose 20.0 g, Monopotassium phosphate 6.0 g, Ammonium citrate 2.0 g, Sodium acetate 25.0 g, Tween 80 1.0 g, Magnesium sulphate 0.575 g, Manganese sulfate 0.12 g, Ferrous Sulfate 0.034 g, Agar 15.0 g, pH5.5
MRSA	Peptone 11.8 g, Yeast dip powder 9.0 g, Lithium chloride 5.0 g, Sodium chloride 55.0 g,

medium (g/L)	Mannitol 10.0 g, Aniline blue 0.2 g, Agar 12.5 g, pH7.2

[1] Vitamins B

Table S2. Actinomycete species identified by culture and/or culture-independent methods

**Actinomycete identified by culture methods**	*Thermoactinomyces vulgaris*	*Thermoactinomyces sacchari*	*Thermoactinomyces intermedius*	*Streptomyces rochei*	*Streptomyces cacaoi*	*Streptomyces zaomyceticus*
**Actinomycete identified by culture-independent methods**	*Thermoactinomyces sanguinis*	*Thermoactinomyces vulgaris*	*Arthrobacter woluwensis*	*Streptomyces albus*	*Kroppenstedtia eburnea*	*Brevibacterium linens*
**Actinomycete undetected with isolation**	*Thermoactinomyces sacchari*	*Streptomyces rochei*	*Streptomyces zaomyceticus*	*Laceyella sacchari*	*Streptomyces griseus*	*Streptomyces sp.* FBKL4.005
**Actinomycete detected without isolation**	*Thermoactinomyces sanguinis*	*Arthrobacter woluwensis*	*Brevibacterium linens*	*Brachybacterium paraconglomeratum*	*Rothia koreensis*	*Streptomyces* NHF165
**Actinomycete Identified by both methods**	*Thermoactinomyces vulgaris*	*Streptomyces albus*	*Thermoactinomyces intermedius*	*Thermostaphylospora chromogena*	*Kroppenstedtiaeburnea*	*Streptomyces cacaoi*

(continued)

Streptomyces griseus	*Streptomyces albus*	*Streptomyces sp.* FBKL4.005	*Thermoactinomyces thalpohillus*	*Streptomyces bangladeshensis*	*Aggregatibacter actinomycetemcomitans*	*Streptomyces flocculus*	*Streptomyces sp.* R11-21
Rothia koreensis	*Streptomyces* NHF165	*Thermoactinomyces intermedius*	*Arthrobacter stackebrandtii*	*Kocuria carniphila*	*Glutamicibacter creatinolyticus*	*Brevibacterium aurantiacum*	*Cellulosimicrobiumfunkei*
Streptomyces bangladeshensis	*Aggregatibacter actinomycetemcomitans*	*Streptomyces flocculus*	*Streptomyces sp.* R11-21	*Massilia timonae*	*Nocardiopsis prasine*	*Streptomyces albofaciens*	*Streptomyces althioticus*
Arthrobacter stackebrandtii	*Kocuria carniphila*	*Glutamicibacter creatinolyticus*	*Brevibacterium aurantiacum*	*Cellulosimicrobium funkei*	*Microbacteriumoxydans*	*Corynebacterium glutamicum*	*Gordoniu terrae*
Streptomyces griseus	*Streptomyces albus*	*Streptomyces sp.* FBKL4.005	*Thermoactinomyces thalpohillus*	*Streptomyces bangladeshensis*	*Aggregatibacter actinomycetemcomitans*	*Streptomyces flocculus*	*Streptomyces sp.* R11-21

(continued)

Thermostaphylospora chromogena	*Massilia timonae*	*Nocardiopsis prasine*	*Streptomyces albofaciens*	*Streptomyces althioticus*	*Streptomyces celluloflavus*	*Streptomyces coelicoflavus*
Microbacterium oxydans	*Corynebacterium glutamicum*	*Gordoniu terrae*	*Dietziamaris*	*Acidipropionibacterium acidipropionici*	*Microbacterium hydrocarbonoxydans*	*Microbacterium schleiferi*
Streptomyces celluloflavus	*Streptomyces coelicoflavus*	*Streptomyces coerulescens*	*Streptomyces cyaneofuscatu s*	*Streptomyces fimicarius*	*Streptomyces flavovirens*	*Streptomyces ghanaensis*
Dietzia maris	*Acidipropionibacter ium acidipropionici*	*Microbacterium hydrocarbonoxydans*	*Microbacteriu m schleiferi*	*Gulosibacter molinativorax*	*Microbacterium hominis*	*Saccharopolyspora rectivirgula*
Thermostaphylospora chromogena	*Massilia timonae*	*Nocardiopsis prasine*	*Streptomyces albofaciens*	*Streptomyces althioticus*	*Streptomyces celluloflavus*	*Streptomyces coelicoflavus*

(continued)

Streptomyces coerulescens	*Streptomyces cyaneofuscatus*	*Streptomyces fimicarius*	*Streptomyces flavovirens*	*Streptomyces ghanaensis*	*Streptomyces griseoplanus*	*Streptomyces halstedii*	*Streptomyces matensis*
Gulosibactermolinati vorax	*Microbacterium hominis*	*Saccharopolyspora rectivirgula*	*Brevibacteriumcel ere*	*Saccharopolyspo ra hordei*	*Kocuriakristinae*	*Marmoricolaaurantia cus*	*Thermoactinomy ces intermedius*
Streptomyces griseoplanus	*Streptomyces halstedii*	*Streptomyces matensis*	*Streptomyces mutabilis*	*Streptomyces olivaceus*	*Streptomyces pactum*	*Streptomyces rubiginosohelvolus*	*Streptomyces sclerotialus*
Brevibacterium celere	*Saccharopolysp ora hordei*	*Kocuria kristinae*	*Marmoricola aurantiacus*	*Thermoactinomy ces intermedius*	*Saccharopolyspo ra hordei*	*Actinopolyspora erythraea*	*Rubrobacter radiotolerans*
Streptomyces coerulescens	*Streptomyces cyaneofuscatus*	*Streptomyces fimicarius*	*Streptomyces flavovirens*	*Streptomyces ghanaensis*	*Streptomyces griseoplanus*	*Streptomyces halstedii*	*Streptomyces matensis*

(continued)

Streptomyces mutabilis	*Streptomyces olivaceus*	*Streptomyces pactum*	*Streptomyces rubiginosohelvolus*	*Streptomyces sclerotialus*	*Streptomyces sindenensis*	*Streptomyces somaliensis*	*Streptomyces thermocarboxyd*

							us
Saccharopolyspora spinosa	*Saccharopolyspora rectivirgula*	*Rubrobacterradiotolerans*	*Streptoalloteichushindustanus*	*Streptomyces coeruleoprunus*	*Aciditerrimonasferrireducens*	*Modestobacter versicolor*	*Nesterenkonia flava*
Streptomyces sindenensis	*Streptomyces somaliensis*	*Streptomyces thermocarboxydus*	*Streptomyces vinaceusdrappus*	*Streptomyces violascens*	*Streptomyces viridobrunneus*	*Streptomyces cinereoruber subsp. cinereoruber*	*Streptomyces ghobisporus subsp. globisporus*
Streptoalloteichus hindustanus	*Streptomyces coeruleoprunus*	*Aciditerrimonas ferrireducens*	*Modestobacter versicolor*	*Nesterenkonia flava*	*Saccharopolyspora spinosa*	*Cutibacterium acnes*	*Corynebacterium sp.* DNF00584
Streptomyces mutabilis	*Streptomyces olivaceus*	*Streptomyces pactum*	*Streptomyces rubiginosohelvolus*	*Streptomyces sclerotialus*	*Streptomyces sindenensis*	*Streptomyces somaliensis*	*Streptomyces thermocarboxydus*

(continued)

Streptomyces vinaceusdrappus	*Streptomyces violascens*	*Streptomyces viridobrunneus*	*Streptomyces cinereoruber subsp. cinereoruber*	*Streptomyces ghobisporus subsp. globisporus*	*Streptomyces diastaticus subsp. ardesiacus*	*Streptomyces lividans*
Ornithinimicrobiumpekingense	*Cutibacterium acnes*	*Corynebacterium sp.DNF00584*	*Streptomyces sp. MG21*	*Actinomycetales sp.JB111*	*Saccharypolyspora rosea*	*Laceyella putida*
Streptomyces diastaticus subsp. ardesiacus	*Streptomyces lividans*	*Streptomyces lividans*	*Nocardiopsis dassonvillei*	*Streptomyces azureus*	*Streptomyces xiamenensis*	*Streptomyces cacaoi subsp. cacaoi*
Streptomyces sp. MG21	*Actinomycetales sp.*JB111	*Saccharypolyspora rosea*	*Laceyella putida*	*Microbispora bispora*	*Streptomyces cacaoi*	*Thermobisporabispora*

Streptomyces vinaceusdrappus	*Streptomyces violascens*	*Streptomyces viridobrunneus*	*Streptomyces cinereoruber subsp. cinereoruber*	*Streptomyces ghobisporus subsp. globisporus*	*Streptomyces diastaticus subsp. ardesiacus*	*Streptomyces lividans*

(continued)

Nocardiopsis dassonvillei	*Streptomyces azureus*	*Streptomyces xiamenensis*	*Streptomyces cacaoi subsp. cacaoi*	*Streptomyces sp.JP12*	*Streptomyces sp.RZ1*	*Micromonospora sp.JD3*	*Arthrobacter protophormiae*
Actinopolyspora erythraea	*Streptomyces cacaoi*	*Thermobispora bispora*	*Thermostaphylospora chromogena*	*Saccharopolyspora erythraea*	*Olsenella uli*	*Olsenella profusa*	*Lancefieldella parvula*
*Streptomyces sp.*JP12	*Streptomyces sp.*RZ1	*Micromonospora sp.*JD3	*Arthrobacter protophormiae*	*Streptomyces roseosporus*	*Streptomyces griseorubroviolaceus*	*Streptomyces aureus*	*Thermophilibacter gallinarum*
Thermostaphylospora chromogena	*Saccharopolyspora erythraea*	*Olsenella uli*	*Olsenella profusa*	*Lancefieldella parvula*	*Corynebacterium tuberculostearicum*	*Corynebacterium minutissimum*	*Streptomyces coeruleorubidus*
Nocardiopsis dassonvillei	*Streptomyces azureus*	*Streptomyces xiamenensis*	*Streptomyces cacaoi subsp. cacaoi*	*Streptomyces sp.JP12*	*Streptomyces sp.RZ1*	*Micromonospora sp.JD3*	*Arthrobacter protophormiae*

(continued)

Streptomyces roseosporus	*Streptomyces griseorubroviolaceus*	*Streptomyces aureus*	*Thermophilibacter gallinarum*	*Streptomyces sampsonii*	*Streptomyces rutgersensis*	*Streptomyces avicenniae*	*Pseudomonas guariconensis*
Corynebacterium tuberculostearicum	*Corynebacterium minutissimum*	*Streptomyces coeruleorubidus*	*Streptomyces hainanensis*				
Streptomyces sampsonii	*Streptomyces rutgersensis*	*Streptomyces avicenniae*	*Arthrobacter aresens*	*Shimazuella kribbensis*	*Kroppenstedtia sanguinis*	*Micrococcus lutens*	*Thermoactinomyces daqus*

Streptomyces hainanensis							
Streptomyces roseosporus	*Streptomyces griseorubroviolaceus*	*Streptomyces aureus*	*Thermophilibacter gallinarum*	*Streptomyces sampsonii*	*Streptomyces rutgersensis*	*Streptomyces avicenniae*	*Pseudomonas guariconensis*

(continued)

Shimazuella kribbensis	*Kroppenstedtia sanguinis*	*Kroppenstedtia eburne*	*Thermoactinomyces daqus*	*Laceyella tengchongensis*	*Laceyella sediminis*	*Laceyella putidus*	*Streptomyces thermoviolaceus*
Laceyella tengchongensis	*Laceyella sediminis*	*Laceyella putidus*	*Streptomyces thermoviolaceus*	*Streptomyces sp.* ZYP3	*Streptomyces sp.* ZYP6	*Streptomyces sp.* ZYP7	*Streptomyces sp.* ZYP10
Shimazuella kribbensis	*Kroppenstedtia sanguinis*	*Kroppenstedtia eburne*	*Thermoactinomycesdaqus*	*Laceyella tengchongensis*	*Laceyella sediminis*	*Laceyella putidus*	*Streptomyces thermoviolaceus*

(continued)

Streptomyces sp. ZYP3	*Streptomyces sp.* ZYP6	*Streptomyces sp.* ZYP7	*Streptomyces sp.* ZYP10	*Streptomyces sp.* ZYP12	*Streptomyces sp.* ZYP13	*Streptomyces sp.* ZYP15	*Streptomyces sp.* ZYP16
Streptomyces sp. ZYP12	*Streptomyces sp.* ZYP13	*Streptomyces sp.* ZYP15	*Streptomyces sp.* ZYP16	*Streptomyces sp.* ZYP17	*Streptomyces sp.* ZYP18	*Streptomyces sp.* ZYP11	*Streptomyces sp.* ZYP9
Streptomyces sp. ZYP3	*Streptomyces sp.* ZYP6	*Streptomyces sp.* ZYP7	*Streptomyces sp.* ZYP10	*Streptomyces sp.* ZYP12	*Streptomyces sp.* ZYP13	*Streptomyces sp.* ZYP15	*Streptomyces sp.* ZYP16

(continued)

Streptomyces sp. ZYP17	*Streptomyces sp.* ZYP18	*Streptomyces sp.* ZYP11	*Streptomyces sp.* ZYP9	*Streptomyces sp.* ZYP8	*Streptomyces sp.* ZYP1	*Streptomyces sp.* ZYP14	*Brevibacteriumrenqingii*

Streptomyces sp. ZYP8	*Streptomyces sp.* ZYP1	*Streptomyces sp.* ZYP14	*Brevibacteriumrenqingii*				
Streptomyces sp. ZYP17	*Streptomyces sp.* ZYP18	*Streptomyces sp.* ZYP11	*Streptomyces sp.* ZYP9	*Streptomyces sp.* ZYP8	*Streptomyces sp.* ZYP1	*Streptomyces sp.* ZYP14	*Brevibacteriumrenqingii*

(continued)

Micrococcus lutens	*Arthrobacter aresens*	*Actinomyces israelii*	*Actinomyces meyeri*	*Bifidobacterium minimum*	*Bifidobacterium magnum*	*Bifidobacterium breve*	*Arthrobacter nicotianae*

(continued)

Nocardia africana	*Nocardia altamirensis*	*Nocardia carnea*	*Nocardia cerradoensis*	*Nocardia flavirosea*	*Nocardia nova*	*Nocardia xishanensis*	

Actinomycete identified by culture methods	Actinomycete identified by culture-independent methods	Actinomycete undetected with isolation	Actinomycete detected without isolation	Actinomycete Identified by both methods
Thermoactinomyces vulgaris	*Thermoactinomyces sanguinis*	*Thermoactinomycessacchari*	*Thermoactinomyces sanguinis*	*Thermoactinomyces vulgaris*

Thermoactinomycessacchari	*Thermoactinomyces vulgaris*	*Streptomyces rochei*	*Arthrobacter woluwensis*	*Streptomyces albus*
Thermoactinomyces intermedius	*Arthrobacter woluwensis*	*Streptomyces zaomyceticus*	*Brevibacterium linens*	*Thermoactinomyces intermedius*
Streptomyces rochei	*Streptomyces albus*	*Laceyellasacchari*	*Brachybacteriumparaconglomeratum*	*Thermostaphylosporachromogena*
Streptomyces cacaoi	*Kroppenstedtiaeburnea*	*Streptomyces griseus*	*Kucuriakoreensis*	*Kroppenstedtiaeburnea*
Streptomyces zaomyceticus	*Brevibacterium linens*	*Streptomyces sp. FBKL4.005*	*Streptomyces NHF165*	*Streptomyces cacaoi*
Laceyellasacchari	*Brachybacteriumparaconglomeratum*	*Thermoactinomycesthalpohillus*	*Thermoactinomyces intermedius*	*Laceyella putida*
Streptomyces griseus	*Kucuriakoreensis*	*Streptomyces bangladeshensis*	*Arthrobacter stackebrandtii*	
Streptomyces albus	*Streptomyces NHF165*	*Aggregatibacteractinomycetemcomitans*	*Kocuriacarniphila*	
Streptomyces sp. FBKL4.005	*Thermoactinomyces intermedius*	*Streptomyces flocculus*	*Glutamicibactercreatinolyticus*	
Thermoactinomycesthalpohillus	*Arthrobacter stackebrandtii*	*Streptomyces sp. R11-21*	*Brevibacteriumaurantiacum*	
Streptomyces bangladeshensis	*Kocuriacarniphila*	*Massiliatimonae*	*Cellulosimicrobiumfunkei*	
Aggregatibacteractinomycetemcomitans	*Glutamicibactercreatinolyticus*	*Nocardiopsis prasine*	*Microbacteriumoxydans*	

Streptomyces flocculus	*Brevibacteriumaurantiacum*	*Streptomyces albofaciens*	*Corynebacterium glutamicum*
Streptomyces sp. R11-21	*Cellulosimicrobiumfunkei*	*Streptomyces althioticus*	*Gordoniu terrae*
Thermostaphylosporachromogena	*Microbacteriumoxydans*	*Streptomyces celluloflavus*	*Dietziamaris*
Massiliatimonae	*Corynebacterium glutamicum*	*Streptomyces coelicoflavus*	*Acidipropionibacteriumacidipr onici*
Nocardiopsis prasine	*Gordoniu terrae*	*Streptomyces coerulescens*	*Microbacteriumhydrocarbonox ydanx*
Streptomyces albofaciens	*Dietziamaris*	*Streptomyces cyaneofuscatus*	*Microbacteriumschleifen*
Streptomyces althioticus	*Acidipropionibacteriumacidipr onici*	*Streptomyces fimicarius*	*Gulosibactermolinativorax*
Streptomyces celluloflavus	*Microbacteriumhydrocarbonox ydanx*	*Streptomyces flavovirens*	*Microbacterium hominis*
Streptomyces coelicoflavus	*Microbacteriumschleifen*	*Streptomyces ghanaensis*	*Saccharopolyspora rectivirgula*
Streptomyces coerulescens	*Gulosibactermolinativorax*	*Streptomyces griseoplanus*	*Brevibacteriumcelere*
Streptomyces cyaneofuscatus	*Microbacterium hominis*	*Streptomyces halstedii*	*Saccharopolyspora hordei*
Streptomyces fimicarius	*Saccharopolyspora rectivirgula*	*Streptomyces matensis*	*Kocuriakristinae*

Streptomyces flavovirens	*Brevibacteriumcelere*	*Streptomyces mutabilis*	*Marmoricolaaurantiacus*
Streptomyces ghanaensis	*Saccharopolyspora hordei*	*Streptomyces olivaceus*	*Thermoactinomycesintermediu s*
Streptomyces griseoplanus	*Kocuriakristinae*	*Streptomyces pactum*	*Saccharopolyspora hordei*
Streptomyces halstedii	*Marmoricolaaurantiacus*	*Streptomyces rubiginosohelvolus*	*Actinopolyspora salina*
Streptomyces matensis	*Thermoactinomycesintermediu s*	*Streptomyces sclerotialus*	*Rubrobacterradiotolerans*
Streptomyces mutabilis	*Saccharopolyspora spinosa*	*Streptomyces sindenensis*	*Streptoalloteichushindustanus*
Streptomyces olivaceus	*Saccharopolyspora rectivirgula*	*Streptomyces somaliensis*	*Streptomyces coeruleoprunus*
Streptomyces pactum	*Rubrobacterradiotolerans*	*Streptomyces thermocarboxydus*	*Aciditerrimonasferrireducens*
Streptomyces rubiginosohelvolus	*Streptoalloteichushindustanus*	*Streptomyces vinaceusdrappus*	*Modestobacter versicolor*
Streptomyces sclerotialus	*Streptomyces coeruleoprunus*	*Streptomyces violascens*	*Nesterenkonia flava*
Streptomyces sindenensis	*Aciditerrimonasferrireducens*	*Streptomyces viridobrunneus*	*Marmoricrobiumpekingense*
Streptomyces somaliensis	*Modestobacter versicolor*	*Streptomyces cinereorubersubspcinereoruber*	*Propionibacterium acnes*

Streptomyces thermocarboxydus	*Nesterenkonia flava*	*Streptomyces ghobisporussubspglobisporus*	*Corynebacterium sp. DNF00584*
Streptomyces vinaceusdrappus	*Ornithinimicrobiumpekingense*	*Streptomycesdiastaticussubsp.ardesiacus*	*Streptomyces sp. MG21*
Streptomyces violascens	*Propionibacterium acnes*	*Streptomyces lividans*	*Actinomycetales sp. JB111*
Streptomyces viridobrunneus	*Corynebacterium sp. DNF00584*	*Streptomyces lividans*	*Saccharypolyspora rosea*
Streptomyces cinereorubersubspcinereoruber	*Streptomyces sp. MG21*	*Nocardiopsisdassonvillei*	*Laceyella putida*
Streptomyces ghobisporussubspglobisporus	*Actinomycetales sp. JB111*	*Streptomyces azureus*	*Microbisporabispora*
Streptomycesdiastaticussubsp.ardesiacus	*Saccharypolyspora rosea*	*Streptomyces xiamenensis*	*Streptomyces cacaoi*
Streptomyces lividans	*Laceyella putida*	*Streptomyces cacaoisubsp.cacaoi*	*Thermobisporabispora*
Nocardiopsisdassonvillei	*Microbisporabispora*	*Streptomyces sp. JP12*	*Thermostaphylosporachromogena*
Streptomyces azureus	*Streptomyces cacaoi*	*Streptomyces sp. RZ1*	*Saccharopolyspora erythraea*
Streptomyces xiamenensis	*Thermobisporabispora*	*Micromonospora sp. JD3*	*Olsenellauli*
Streptomyces cacaoisubsp.cacaoi	*Thermostaphylosporachromogena*	*Arthrobacter protophormiae*	*Olsenella umbonate*

Streptomyces sp. JP12	*Saccharopolyspora erythraea*	*Streptomyces roseosporus*	*Atopobiumparvuium*
Streptomyces sp. RZ1	*Olsenellauli*	*Streptomyces griseorubroviolaceus*	*Corynebacterium tuberculostearicum*
Micromonospora sp. JD3	*Olsenella umbonate*	*Streptomyces aureus*	*Corynebacterium minutissimum*
Arthrobacter protophormiae	*Atopobiumparvuium*	*Thermophilibactergallinarum*	*Streptomyces coeruleorubidus*
Streptomyces roseosporus	*Corynebacterium tuberculostearicum*	*Streptomyces sampsonii*	*Streptomyces hainanensis*
Streptomyces griseorubroviolaceus	*Corynebacterium minutissimum*	*Streptomyces rutgersensis*	*Saccharopolyspora spinosa*
Streptomyces aureus	*Streptomyces coeruleorubidus*	*Streptomyces avicenniae*	
Thermophilibactergallinarum	*Streptomyces hainanensis*	*Arthrobacter aresens*	
Streptomyces sampsonii	*Actinopolyspora salina*	*Shimazuellakribbensis*	
Streptomyces rutgersensis		*Kroppenstedtia sanguinis*	
Streptomyces avicenniae		*Micrococcus lutens*	
Pseudomonas guariconensis		*Thermoactinomycesdaqus*	

Shimazuellakribbensis	*Laceyellatengchongensis*
Kroppenstedtia sanguinis	*Laceyellasediminis*
Kroppenstedtiaeburne	*Laceyellaputidus*
Thermoactinomycesdaqus	*Streptomyces thermoviolaceus*
Laceyellatengchongensis	*Streptomyces sp. ZYP3*
Laceyellasediminis	*Streptomyces sp. ZYP6*
Laceyellaputidus	*Streptomyces sp. ZYP7*
Streptomyces thermoviolaceus	*Streptomyces sp. ZYP10*
Streptomyces sp. ZYP3	*Streptomyces sp. ZYP12*
Streptomyces sp. ZYP6	*Streptomyces sp. ZYP13*
Streptomyces sp. ZYP7	*Streptomyces sp. ZYP15*
Streptomyces sp. ZYP10	*Streptomyces sp. ZYP16*
Streptomyces sp. ZYP12	*Streptomyces sp. ZYP17*
Streptomyces sp. ZYP13	*Streptomyces sp. ZYP18*

Streptomyces sp. ZYP15	*Streptomyces sp. ZYP11*
Streptomyces sp. ZYP16	*Streptomyces sp. ZYP9*
Streptomyces sp. ZYP17	*Streptomyces sp. ZYP8*
Streptomyces sp. ZYP18	*Streptomyces sp. ZYP1*
Streptomyces sp. ZYP11	*Streptomyces sp. ZYP14*
Streptomyces sp. ZYP9	*Brevibacteriumrenqingii*
Streptomyces sp. ZYP8	
Streptomyces sp. ZYP1	
Streptomyces sp. ZYP14	
Brevibacteriumrenqingii	
Micrococcus lutens<br>*Arthrobacter aresens*	
Actinomyces israelii	
Actinomyces meyeri	

Bifidobacterium minimum
Bifidobacterium magnum
Bifidobacterium breve
Arthrobacter nicotianae
Nocaridiaafricana
Nocaridiaaltamirensis
Nocaridiacarnea
Nocaridiacerradoensis
Nocaridiaflavirosea
Nocaridia nova
Nocaridia soli
